# Disordered animal multilayer reflectors and the localization of light

**DOI:** 10.1098/rsif.2014.0948

**Published:** 2014-12-06

**Authors:** T. M. Jordan, J. C. Partridge, N. W. Roberts

**Affiliations:** 1 School of Biological Sciences, University of Bristol, Bristol Life Sciences Building, Tyndall Avenue, Bristol BS8 1TQ, UK; 2 Bristol Centre for Complexity Sciences, University of Bristol, Queens Building, University Walk, Bristol BS8 1TR, UK; 3 School of Animal Biology and the Oceans Institute, Faculty of Science, University of Western Australia, 35 Stirling Highway (M317), Crawley, Western Australia 6009, Australia

**Keywords:** disordered photonics, Anderson localization, biophotonics, structural colour, broadband reflectivity, polarization-insensitive reflectivity

## Abstract

Multilayer optical reflectors constructed from ‘stacks’ of alternating layers of high and low refractive index dielectric materials are present in many animals. For example, stacks of guanine crystals with cytoplasm gaps occur within the skin and scales of fish, and stacks of protein platelets with cytoplasm gaps occur within the iridophores of cephalopods. Common to all these animal multilayer reflectors are different degrees of random variation in the thicknesses of the individual layers in the stack, ranging from highly periodic structures to strongly disordered systems. However, previous discussions of the optical effects of such thickness disorder have been made without quantitative reference to the propagation of light within the reflector. Here, we demonstrate that Anderson localization provides a general theoretical framework to explain the common coherent interference and optical properties of these biological reflectors. Firstly, we illustrate how the localization length enables the spectral properties of the reflections from more weakly disordered ‘coloured’ and more strongly disordered ‘silvery’ reflectors to be explained by the same physical process. Secondly, we show how the polarization properties of reflection can be controlled within guanine–cytoplasm reflectors, with an interplay of birefringence and thickness disorder explaining the origin of broadband polarization-insensitive reflectivity.

## 1. Introduction

The application of physical theory to optical structures in animals has a long history. Beginning with models of thin-film interference in the early twentieth century [1] and periodic multilayer reflectors in the 1960s and 1970s [2–4], through to photonic crystals [5–7], quasi-ordered amorphous solids [8,9] and incoherent scattering structures [10] in recent years. The ongoing physical and mathematical characterization of these structures has provided great insight into a variety of biological topics, including crypsis strategies [11,12], intraspecific communication [13–15] and adaptations in eye designs that require the reflection of light [2,16,17]. Furthermore, in certain cases, optical structures in animals provide mechanisms that are of interest for both replication in optical technologies [18,19] and bioinspired theoretical analysis [8,20].

Animal reflective structures are described as being a ‘multilayer’ when they are organized into an approximately layered, one-dimensional ‘stack’ geometry. In the skin and scales of fish [3,21–24] and the eyes of spiders [16,25] the layers are guanine crystals with cytoplasm gaps, in the iridophores of cephalopods the layers are protein platelets with cytoplasm gaps [3,26,27] and in butterfly wings the layers are chitin and air [4,28]. By controlling the values and the distribution of the layer thicknesses in the reflector, animals are able to produce both narrowband ‘coloured’ reflectivity (where the high- and low-index layers have approximately the same thicknesses throughout the near-periodic structure [2–4,26,27,29]) and broadband ‘silver’ reflectivity (where the high- and low-index layers have randomly distributed thicknesses about a mean value [3,21,22,24,30]).

Animal multilayer reflectors that are approximated as periodic can be theoretically characterized using a ‘quarter-wave stack’ analytical model in which both the high- and low-index layers in the reflector have optical thickness equal to a quarter of the peak reflection wavelength [3,4,31]. In addition, a modern analogy is sometimes drawn between periodic animal multilayer reflectors and one-dimensional photonic crystals [32,33]. The spectral bandwidth of the high reflection region is associated with the ‘photonic band-gap’, which describes the spectral region where light cannot propagate within the structure [34,35]. By contrast, the theoretical characterization of the reflectivity from animal reflectors that contain a higher level of disorder cannot be approximated to a ‘quater-wave stack’. Calculations of the reflectivity have been reliant upon numerical modelling, and consequently, some commonly occuring optical properties, such as the presence of unbroken broadband ‘silvery’ reflection spectra [3,21,22,24] or polarization-insensitive reflectivity [13,22,36], lack an explanation in terms of the propagation of light within the reflective structure.

A physical parallel between random stack models of animal multilayer reflectors and Anderson localization has been suggested in two previous biophotonic studies [20,21], although has yet to be explored in any detail. The theory of Anderson localization explains how waves become spatially confined in a disordered medium. It was originally conceived as a way to explain the transport properties of electrons in a semiconductor and the related behaviour of the quantum wave function [37]. The theory is now, however, understood to be a universal wave phenomenon that also applies to electromagnetic waves [38–40], matter waves [41] and acoustic waves [42]. The physical origin of Anderson localization is entirely due to multiple scattering and coherent interference [40]. In one-dimensional random stack systems (which includes optical multilayer reflectors), the theory of Anderson localization predicts an exponential decay in the amplitude of the transmitted wave as a function of the system length; an effect that is quantified by the localization length [43,44]. In random optical multilayers, the exponential decay in transmission provides a general explanation for the production of broadband mirror-like reflectivity [45].

In this paper, we illustrate that the theory of Anderson localization and the property of the localization length enables the reflectivity from animal multilayer reflectors with varying degrees of disorder to be understood within a common theoretical framework. Our paper should not be seen as a demonstration of a new way of calculating reflection spectra, more an illustration that a diversity of optical properties (including ‘coloured’, ‘silvery’ and polarization-insensitive reflectivity) can be explained by the same coherent interference process. We summarize the trends in layer thickness disorder in animal reflectors (§2), and then describe how the reflectivity and localization length can be calculated (§3). We then illustrate how, from the perspective of localization theory, disordered animal multilayer reflectors can control the spectral properties (§4) and the polarization properties (§5) of reflection. Finally, we discuss the consequences of our study for both biologists and physicists (§6).

## 2. Thickness disorder in animal multilayer reflectors

**Figure 1.**
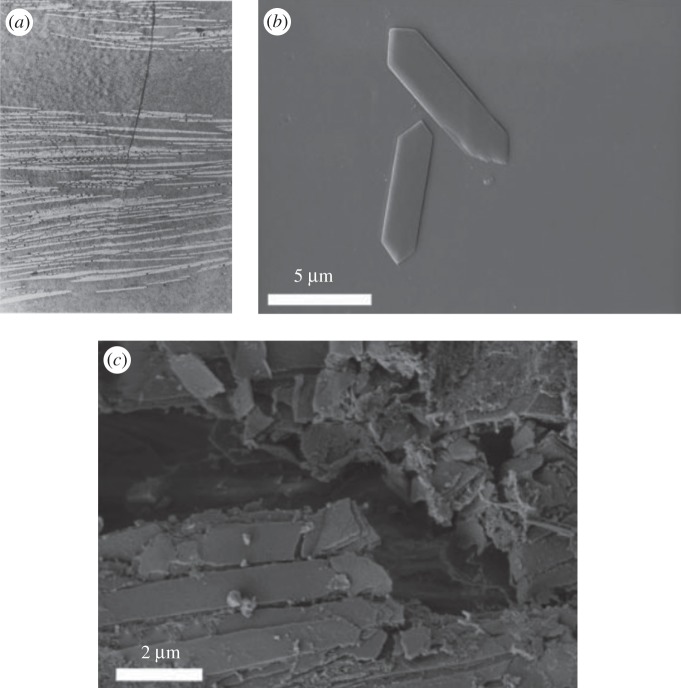
(*a*) A transmission electron microscopy section of a disordered guanine–cytoplasm multilayer reflector in the skin of *Lepidoptus caudatus* [21]. (*b*) An individual guanine crystal in solution from *Cyprinus carpio* [23]. (*c*) An individual guanine crystal *in situ* from *Cy. carpio* [23].

Throughout this paper, we use guanine–cytoplasm reflectors (common to fish and spiders) as a model system. These reflectors have been well described in the previous literature [3,16,21–25]. Figure 1*a* is a transmission electron micrograph from *Lepidoptus caudatus* (silver scabbard fish), reproduced from [21], that shows the guanine crystals (the lighter streaks) and cytoplasm gaps (the darker surrounding media) that form a typical reflector. The variation in both the thickness and spacing of the crystals is illustrated. Figure 1*b* further illustrates the shape and form of isolated individual guanine crystals in solution (reproduced from [23]), and figure 1*c in situ* in the skin of *Cyprinus carpio* (Japanese Koi; also reproduced from [23]).

In the random stack representation of guanine–cytoplasm reflectors, it is the mean, standard deviation and probability distribution of the thickness of the layers (along with their dielectric properties which are discussed in §3) that define the reflector [21]. Table 1 summarizes layer thicknesses for a range of guanine–cytoplasm reflectors from the literature and includes reflectors in fish skin: *L. caudatus* [21], *Clupea harengus* (Atlantic herring) [22], *Cy. carpio* [24] and *Pentapodus paridiseus* (paradise whiptail) [29]; fish scales: *Sprattus sprattus* (European sprat) [3]; mollusc eyes: *Pecten maximus* (king scallop) [2]; spider eyes: *Drassodes cupres* (ground spider) [16] and spider skin: *Tetragnatha extensa* (common stretch-spider) [24]. Where possible the mean thicknesses, 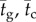, standard deviations, *σ*_g_, *σ*_c_, and the probability distributions for the guanine crystal and the cytoplasm gap thicknesses are provided. The thicknesses in table 1 have been measured using a variety of techniques including transmission electron microscopy [16,21], scanning electron microscopy [24] and optical interference microscopy [3].

**Table 1.** Summary of the layer thicknesses, total lengths and number of crystal layers for guanine–cytoplasm animal multilayer reflectors. The mean values and standard deviations for the guanine and cytoplasm layers are, 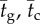, *σ*_g_,*σ*_c_, respectively. The relative standard deviations (RSD) are 
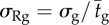
, 
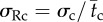
. The total length refers to the length of the reflecting stack.

animal	description of reflector	guanine thickness ( 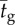 , *σ*_g_, distribution)	cytoplasm thickness ( 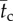 , *σ*_c_, distribution)	guanine RSD 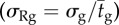	cytoplasm RSD 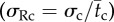	total length	number of guanine layers
*Lepidopus caudatus* (ribbon fish) [21]	silvery reflector in skin	110 ± 32 nm (uniform)	160 ± 44 nm (uniform)	0.29	0.28	27 µm	100
*Clupea harengus* (Atlantic herring) [22]	silvery reflector in *stratum argenteum*	83 ± 16 nm (uniform)	165 ± 78 nm (uniform)	0.19	0.47	8.4 µm	37
*Cyprinus carpio* (carp) [24]	reflector in iridophore	19 ± 6 nm (normal)	231 ± 94 nm (normal)	0.32	0.41	7.5 µm	30
*Sprattus sprattus* (European sprat) [3]	coloured reflector in scale	95 ± 8 nm (normal)	not given	0.08	—	1 µm	6
*Pentapodus paridiseus* (paradise whiptail) [29]	blue reflector in iridophore	52 ± 4 nm (normal)	84 ± 12 nm (normal)	0.08	0.14	1.3 µm	10
*Pecten maximus* (king scallop) [2]	reflector in eye	73 ± 5 nm (normal)	not given	0.07	—	6–8 µm	30–40
*Drassodes cupres* (ground spider) [16]	reflector in eye	58 ± 12 nm (normal)	115 ± 37 nm (normal)	0.21	0.32	2.6–3.5 µm	15–20
*Tetragnatha extensa* (king spider) [24]	reflector in skin	19 ± 6 nm (normal)	203 ± 59 nm (normal)	0.32	0.29	not given	not given

Data from [2,16,22,24,29] are taken directly from values quoted in the text of each paper, whereas data from [3,21] are estimated from histograms presented in each paper. The studies of [21,22] are for random stack models of uniformly distributed layer thicknesses about a mean value, which has standard deviation defined by2.1
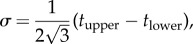
where *t*_upper_ and *t*_lower_ are the upper and lower bounds on the maximum and minimum layer thicknesses, respectively. The other studies are for thicknesses that are normally distributed about a mean value. Table 1 includes relative standard deviation values, 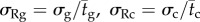 which provide dimensionless and comparative measures of the thickness disorder for the high and low refractive index layers in the different animal reflectors.

Table 1 also includes an estimate of the total length and number of crystal layers/periods in each reflector. These values are approximate, as the number of crystals often varies between different regions of tissue (e.g. 30–40 crystal layers for *Pecten maximus* [2]).

**Figure 2.**
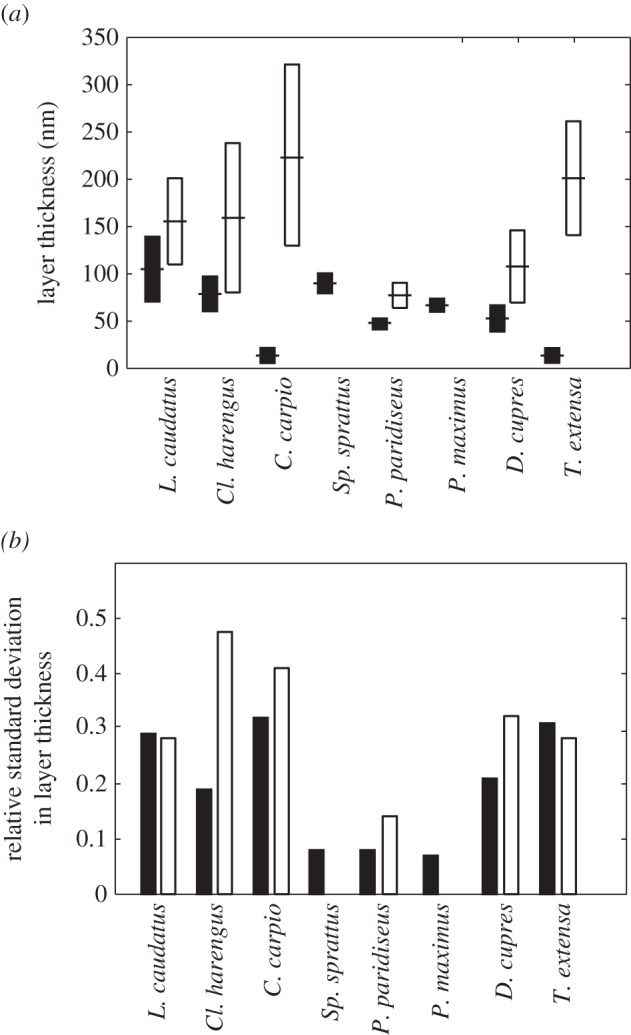
Mean, standard deviation, and relative standard deviation of layer thicknesses for guanine–cytoplasm animal multilayer reflectors. (*a*) Mean values, 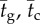, (central dashes) and standard deviations, *σ*_g_, *σ*_c_, (bar limits), (*b*) Relative standard deviations, 
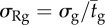
, 
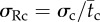
. In both (*a*,*b*), the guanine layers are the left black bars and the cytoplasm layers are the right white bars. Data are not available for the cytoplasm layers in *Sp. sprattus* and *P. maximus*. Layer thicknesses are uniformly distributed in the cases of *L. caudatus* and *Cl. harengus* and are normally distributed (or presumed so, see table 1) in all other cases.

The mean layer thicknesses, 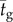, 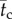, and standard deviations, *σ*_g_, *σ*_c_, for the animal multilayer reflectors in table 1 are illustrated graphically in figure 2*a*, and the corresponding relative standard deviations 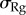, 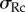 are shown in figure 2*b*. Common to each reflector is that both the mean thickness and standard deviation of the guanine crystal layers is lower than the respective values for the cytoplasm gaps. This is especially apparent for the reflectors in *Cy. carpio* and *T. extensa*. By contrast, the *σ*_R_ values for the guanine and cytoplasm in each reflector are much more similar. The reflectors in *L. caudatus*, *Cl. harengus*, *Cy. carpio*, *D. cupres*, *T. extensa* have *σ*_Rg_ and *σ*_Rc_ values that are typically in the range 0.25–0.40, whereas the reflectors in *Sp. sprattus*, *P. maximus*, *P. paridiseus* have *σ*_Rg_ and *σ*_Rc_ values approximately 0.1. Data for the cytoplasm gap thicknesses in the more weakly disordered structures are not generally presented explicitly in the literature. However, the initial optical characterization of these structures established that the thickness disorder in the cytoplasm layers must be fairly small to be able to produce the near-ideal quarter-wave reflection behaviour that is observed [2–4]. In general, more strongly disordered reflectors are of greater total length than less disordered reflectors.

## 3. Calculating the reflectivity and localization length

### 3.1. Transfer matrix models

The reflection and transmission of light in multilayer systems (random or periodic) can be calculated using optical transfer matrix methods [46–48]. The reflection (reflectivity) and transmission (transmissivity) coefficients are a function of wavelength, angle of incidence and polarization and notated by *R_s_*(*λ*,*θ*), *R_p_*(*λ*,*θ*), *T_s_*(*λ*,*θ*), *T_p_*(*λ*,*θ*), where *λ* is the wavelength of light in a vacuum, *θ* is the angle of incidence and the subscripts *s* and *p* refer to the polarization mode (light linearly polarized perpendicular and parallel to the plane of incidence, respectively). Transfer matrix models naturally incorporate the multiple scattering and coherent interference that is necessary for Anderson localization to occur [40,49].

The vast majority of transfer matrix models of animal multilayer reflectors have assumed that the layers are dielectrically isotropic and a classic treatment of this method is provided in [46]. Isotropic transfer matrix models of guanine–cytoplasm reflectors typically assume that the refractive index of guanine is *n*_g_ = 1.83 [4,21,24]. The refractive index used for the cytoplasm layers is typically *n*_c_ = 1.33 [4,21,22] (i.e. non-dispersive and approximated to be the same as water). This value is assumed for both the cytoplasm layers and the external media in this paper.

Despite widespread use of isotropic models, guanine crystals, which are principally composed of the purine guanine and the purine-derivate hypoxanthine [50], are highly birefringent [11,22,23,50]. The crystals are weakly biaxial and have principle refractive indices of (1.85, 1.81, 1.46) [11,50]. A uniaxial approximation of the guanine crystals in which the principle refractive indices are (1.83, 1.83, 1.46) based upon the general 4 × 4 transfer method for stratified anisotropic media [47,48] is provided in [22] and is used in this paper. In general, the guanine crystal layers can have different orientations of their principle axes relative to the stack coordinate system [22]. These optically distinct birefringent stack layers are defined as *Type 1* and *Type 2* crystals in [22] and we use the same notation here. The refractive index vectors in the Cartesian system (which has the direction of stacking aligned with the *z*-axis) are given by3.1
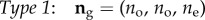
and3.2
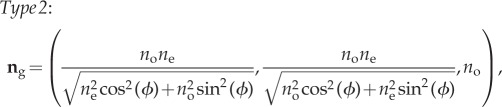
where *n*_o_ = 1.83 is the ordinary refractive index (i.e. taken to be the same value as *n*_g_ in the isotropic model), *n*_e_ = 1.46 is the extraordinary refractive index and *ϕ* is a rotation angle in the (*x*, *y*)-plane. *Type 1* crystals correspond to a uniaxial model of the most commonly reported type of guanine crystal (e.g. [11,23,50]), whereas *Type 2* crystals are a uniaxial model of some of the guanine crystals that occur in the *stratum argentem* (a sub-dermal layer) of *Cl. harengus*, *Sp. sprattus* and *Sardina pilchardus* (European sardine) [22].

Transfer matrix models of guanine–cytoplasm reflectors typically assume that the layers are optically transparent in the animal-visible region of the spectrum (approx. 300–800 nm) and that they are non-magnetic and non-dispersive. Optical transparency is a very good approximation for models of guanine–cytoplasm reflectors in the optical region as the absorption spectrum for guanine has peaks in the UV region *λ* < 300 nm [51], and the absorption spectrum for cytoplasm/water has peaks in the infrared region *λ* > 1400 nm [21].

### 3.2. Spatial averaging of reflectivity spectra

The early models of periodic quarter-wave animal reflectors are based upon a single set of physical parameters or ‘stack configuration’ [3,4,31]. However, when optically modelling the reflectivity of animal multilayer structures with thickness disorder, it is common to ensemble average the reflectivity spectra over a set of random stack configurations each of which has the layer thicknesses sampled from a probability distribution [21,22,24,26,30].

**Figure 3.**
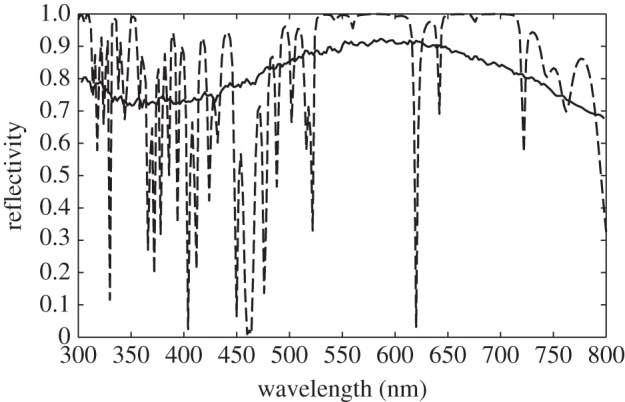
Reflectivity spectra from a random stack model of a disordered animal multilayer reflector. The reflectivity spectrum of an ensemble (spatial) average, 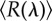, is indicated by the solid black line, and the spectrum of a single random stack configuration, *R*(*λ*), is indicated by the dashed black line. This plot is for the model system described in §4 with *σ*_R_ = 0.4, and the ensemble average uses 500 random stack configurations.

Example reflectivity spectra for an ensemble average, 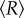, and a single random stack configuration, *R*, are shown in figure 3. The ensemble averaged spectrum represents a spatial averaging of the optically modelled reflectivity and enables a comparison to be made with the experimentally measured ‘macroscopic’ reflectivity [21,22]. For spatial averaging to occur, the planar projection of a typical guanine crystal must be several orders of magnitude smaller than an experimental beam spot size. This is likely to be the case as, assuming a 1 mm beam spot radius, and planar crystal dimensions of approximately 25 × 5 µm (based on data from *Sp. sprattus* [3]), there are approximately 2.5 × 10^4^ stack configurations in a ‘macroscopic’ reflection measurement. The spatial averaging implicitly assumes that the average structure is homogeneous throughout the reflecting surface. The single random stack configuration exhibits sharp gaps in the reflectivity spectrum which in localization theory are referred to as transmission resonances [44]. These resonant features do not occur in experimental measurements from animal multilayer reflectors [21,22,30] principally because of the physical scale at which such measurements have been made.

### 3.3. Calculating the localization length

The properties and criteria for Anderson localization to occur depend upon the dimension of the system under consideration [39,52]. In this paper, we are entirely concerned with the properties of localization in one dimension, where a general mathematical theorem demonstrates that all waves in one dimension are localized and that the amplitude of the transmitted wave decreases exponentially with the length of the system [53,54]. For electromagnetic waves, this leads to the following definition of the localization length:3.3
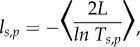
where *L* is the reflector length and 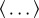 denotes ensemble average [43]. It is clear that, as is the case for the stack transmissivity, the localization length is a function of angle of incidence, wavelength and polarization. A physical explanation of how the exponential decay in transmissivity in equation (3.3.) arises entirely from coherent thin-film interference is provided by Berry & Klein [45]. Localization of electromagnetic waves occurs when there is random layer thickness and/or random dielectric permittivity/refractive index values for the layers in the stack [49,55].

**Figure 4.**
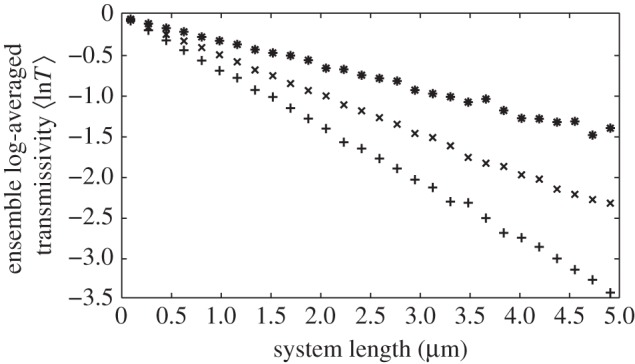
The exponential decay in ensemble log-averaged transmissivity. The black circles are for *λ* = 400 nm, the black oblique crosses are for *λ* = 500 nm, and the black cardinal crosses are for *λ* = 600 nm. The gradients are equal to 2/*l*, where *l* is the localization length, and correspond to *l* = 6.65, 4.17 and 2.94 µm for *λ* = 400, 500, 600 nm, respectively. All plots are for the model described in §5 with *σ*_R_ = 0.4 for both high- and low-index layers. The simulations were averaged over 500 stack configurations.

By performing log-linear regression of equation (3.3), it is possible to calculate numerically the localization length as a function of wavelength, angle of incidence and polarization [49,56]. An example is illustrated in figure 4, which demonstrates the exponential decay in log-averaged transmissivity with system length at different wavelengths for the model considered in §4. The gradients in figure 4 are equal to 2/*l*, and correspond to *l* = 6.65, 4.17, 2.94 µm at *λ* = 400, 500, 600 nm, respectively. Note, that logarithmic averaging of the transmissivity is used in equation (3.3). The reason for this is that the log-average is a ‘self-averaging’ quantity (i.e. that the log-average in a finite system is representative of a single infinite system [57]). The ensemble averaging of transmissivity and reflectivity (which corresponds to the spatial average in random stack models on animal reflectors) are not self-averaging [44]. In this paper, however, we do not dwell on this technical subtlety, and we assume that the spatial average of the reflectivity well describes the ‘average’ reflection properties of disordered animal multilayer reflectors.

## 4. Localization and control over the spectral properties of reflection

### 4.1. Overview of the model

Anderson localization provides a means of understanding how animal multilayer reflectors can control the spectral bandwidth of their high reflection region through different degrees of thickness disorder. This control over the apparent colour of reflection is thus related to the thickness measurements described in §2. It is important to highlight the difference in the transmission of light in stack systems for two limiting physical regimes of thickness disorder. Firstly, for the no disorder limit of periodic multilayer reflectors (one-dimensional photonic crystals) the exponential decay in transmissivity only occurs in the band-gap regions [58,59]. Subsequently, the regions of high reflectivity only occur in narrow spectral intervals resulting in a coloured appearance for animal multilayer reflectors [4,32]. Secondly, in the mathematically ideal case of strong disorder (where the wave phases in the reflector are entirely random) the exponential decay in transmissivity is wavelength-independent, resulting in a wavelength-independent formula for the localization length [45,60]. Subsequently, the reflectivity for the ideal case of strong disorder is also wavelength-independent. The levels of disorder in the thicknesses of the animal multilayer reflectors described in §2 fall between these two limiting regimes, and here we quantify how the localization length relates to the reflectivity and apparent colour of these systems.

The model set out below considers random perturbations to the layer thicknesses around an ‘underlying’ periodic quarter-wave stack structure and is analogous to systems described in classical optics literature [55,61,62]. The model is for *Type 1* guanine crystal layers, defined in [22] and equation (3.1), which for normal incidence is equivalent to isotropic guanine crystal layers with *n*_o_ = *n*_g_. When describing this model, we interchangeably use the number of crystal layers *N* and the system length *L* = *Na*_0_ where4.1
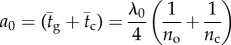
is the mean periodicity of the system, with 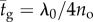 and 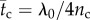 the mean layer thicknesses governed by the quarter-wave condition, and *λ*_0_ the wavelength of peak reflectivity. The value *λ*_0_ = 550 nm is used as it is approximately the centre of the spectral region that is relevant to animal visual systems [63].

It is convenient to use the relative standard deviation of layer thickness, *σ*_R_, to parametrize the thickness disorder. This is chosen to be the same for both the guanine and cytoplasm layers in the reflector that captures the general trend in thickness disorder described in §2. This model also has the advantage of reducing the layer thickness disorder to a single free parameter. The disorder is introduced by considering perturbations to the layer thickness of the form4.2
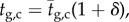
where *δ* is uniformly distributed on the interval 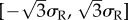. The choice of uniform probability distribution follows from the models in [21,22]. It is more practical for numerical simulation than an (unbounded) normal distribution, which can lead to the sampling of negative layer thickness. The definition of the bounds upon *δ* can, however, be approximately related to the values of *σ*_R_ for the physical data in figure 2, and from equation (2.1) the respective bounds upon the layer thicknesses are given by 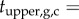 
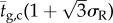
 and 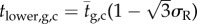.

### 4.2. Localization and reflectivity spectra

**Figure 5.**
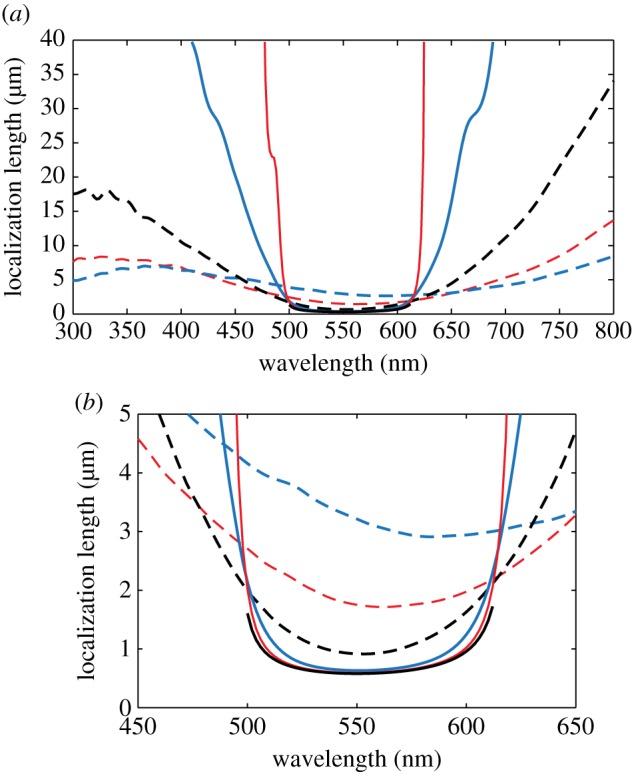
Localization length spectra at normal incidence for different levels of layer thickness disorder. Panel (*a*) is over the full animal-visible spectral range and (*b*) is a detail plot of the band-gap region of the underlying quarter-wave structure. The layer thickness disorder is parametrized by the relative standard deviation in layer thickness, *σ*_R_, which is the same for high- and low-index layers. The parameter sets are identical in each plot with the red solid lines for *σ*_R_ = 0.05, the blue solid lines for *σ*_R_ = 0.1, the black long dashed lines for *σ*_R_ = 0.2, the red long dashed lines for *σ*_R_ = 0.3 and the blue long dashed lines for *σ*_R_ = 0.4. The solid black lines represent a lower bound upon the localization length and are calculated using the transmissivity of the underlying periodic quarter-wave structure in the band-gap region. The simulations were averaged over 500 stack configurations.

**Figure 6.**
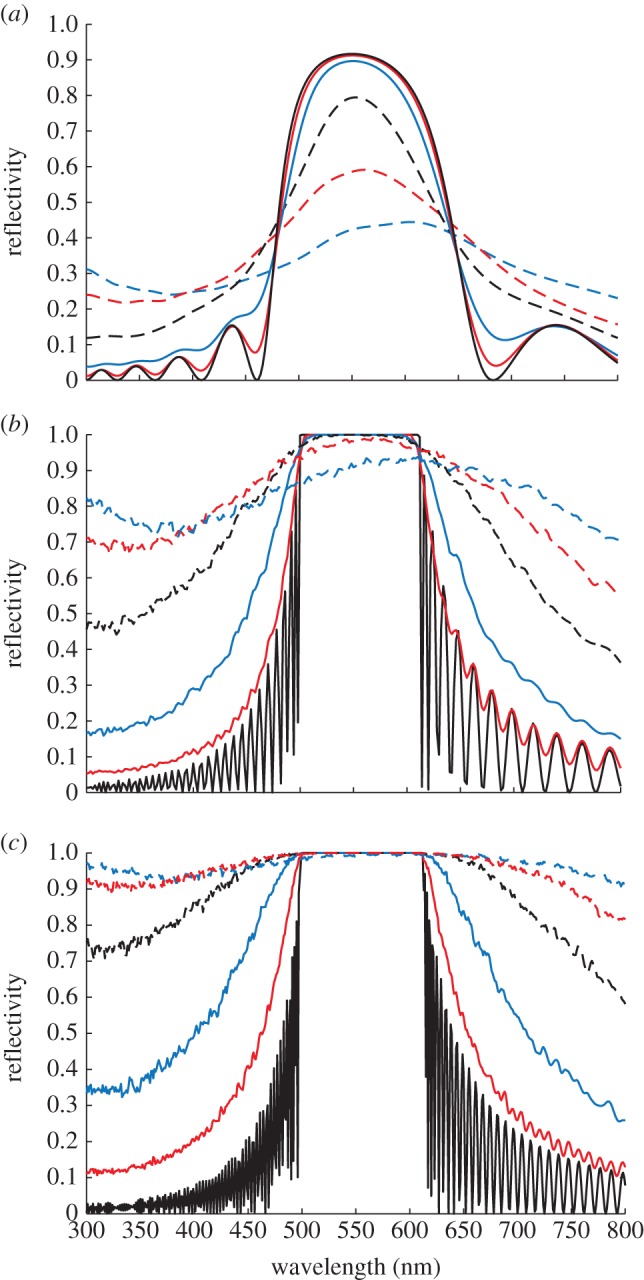
Reflectivity spectra of a randomly perturbed quarter-wave stack at normal incidence for different levels of layer thickness disorder. (*a*) *N* = 6, *L* = 0.89 µm, (*b*) *N* = 40, *L* = 7.05 µm and (*c*) *N* = 100, *L* = 17.6 µm, where *N* is the number of crystal layers/periods and *L* is the length of the reflector. The layer thickness disorder is parametrized by the relative standard deviation in layer thickness, *σ*_R_, which is the same for high- and low-index layers. In all plots, the red solid lines are for *σ*_R_ = 0.05, the blue solid lines are for *σ*_R_ = 0.1, the black long dashed lines are for *σ*_R_ = 0.2, the red long dashed lines are for *σ*_R_ = 0.3 and the blue long dashed lines are for *σ*_R_ = 0.4. The black solid lines are for the underlying quarter-wave structures with peak reflectivity at *λ*_0_ = 550 nm. The simulations were averaged over 500 stack configurations. The plot illustrates the general trend that the width of the high reflection region increases as the level of disorder increases.

Localization length spectra for values of *σ*_R_ that range from 0.05 to 0.40 (which broadly represents the range of thickness disorder described in §2) are shown in figure 5. The band-gap region of the underlying periodic stack has upper and lower wavelength limits of 500 nm and 612 nm, respectively, which can be calculated using formulae supplied in [59]. The lower bound upon the localization length in the band-gap region (indicated by the solid black lines) is calculated using the transmissivity of the underlying periodic structure. Corresponding ensemble averaged reflectivity spectra are shown in figure 6, for *N* = 6, 40, 100 crystal layers/periods, which from equation (4.1) corresponds to lengths of *L* = 0.89, 7.05, 17.6 µm. The values are based upon the typical number of crystal layers in *Sp. sprattus*, *Cl. harengus* and *L. caudatus* in table 1 but differ slightly due to the standardized periodicity, *a*_0_.

For weakly disordered animal multilayer reflectors where *σ*_R_ = 0.05, 0.10 (this roughly corresponds to the thickness disorder for the reflectors in *Sp. sprattus*, *P. maximus*, *P. paridiseus* described in §2), the localization length is drastically shorter in the band-gap region than outside. The result of this strong spectral dependence to the localization length is that the ensemble averaged reflectivity spectra for *σ*_R_ = 0.05, 0.10 are very similar to the underlying periodic structure. This analysis underpins the previous work on quarter-wave stacks [2,3,4,26,29], where it is generally implied that the thickness disorder in these animal multilayer reflectors is sufficiently small to not significantly affect the ‘ideal’ quarter-wave behaviour. Another feature evident in figure 6, which has also been noted in past studies [3,26], is that the high-frequency oscillations outside the band-gap region for the periodic quarter-wave structures (the solid black lines in figure 6) are ‘smoothed out’ by the presence of thickness disorder.

**Figure 7.**
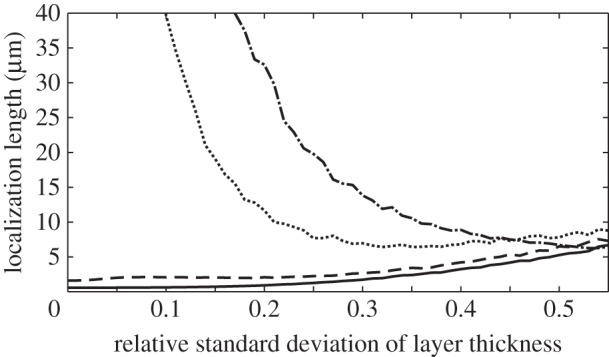
Localization length as a function of the relative standard deviation of layer thickness, *σ*_R_, for different wavelengths. The black solid line is for *λ* = 550 nm (approximately the wavelength centre of the band-gap region of the underlying quarter-wave structure), the black long dashed line is for *λ* = 500 nm (at the edge of the band-gap region of the underlying quarter-wave structure), the black dotted line is for *λ* = 400 nm and the black tray dashed line is for *λ* = 800 nm (both outside the band-gap region of the underlying quarter-wave structure). The convergence of all lines at the right of the plot demonstrates the transition from strong to weak spectral dependence of the localization length as the layer thickness disorder increases.

For a given reflector length, increasing the degree of layer thickness disorder has the overall effect of increasing the values of the reflectivity outside the band-gap region, and decreasing the values of the reflectivity inside (figure 6). This optical behaviour can be related to the properties of the localization length (figure 5), which undergo a transition from strong to weak spectral dependence as the thickness disorder increases. Figure 7 shows the localization length as an explicit function of *σ*_R_ at different wavelengths, corresponding to *λ* = 550 nm (the peak reflectivity value of the underlying quarter-wave structure, and close to the wavelength centre of the band-gap), *λ* = 500 nm (at the edge of the band-gap region of the underlying quarter-wave structure) and *λ* = 400 and 800 nm (both outside the band-gap region of the underlying quarter-wave structure). For *σ*_R_ > 0.3, the localization length in the different spectral regions is less than 13 µm and for *σ*_R_ ∼ 0.5 the localization length values in the different spectral regions have approximately converged in the range approximately 5–8 µm. This near-wavelength independence of the localization length provides a structural justification for how the more disordered animal multilayer reflectors in §2 (*L. caudatus*, *Cy. carpio* and *Cl. harengus*) are able to produce spectrally ‘flat’ broadband reflectivity across the animal-visible region of the spectrum, resulting in a ‘silvered’ appearance.

## 5. Localization and control over the polarization properties of reflection

### 5.1. Overview of model

Guanine–cytoplasm reflectors can also produce both polarizing and polarization-insensitive reflectivity, thus controlling the polarization properties of reflection. Here, we describe the very general relationships that occur between the localization length, the mean (polarization averaged) reflectivity and the reflectivity of the *s*- and *p*- polarization modes in broadband polarizing and polarization-insensitive reflectors. The model used in this section is based upon previous analysis of guanine–cytoplasm reflectors in the *stratum argenteum* of *Cl. harengus* and *Sa. pilchardus* [22]. In the general model, the reflectors contain a mixture of *Type 1* and *Type 2* crystals (equations (3.1) and (3.2)), and as portrayed in a schematic diagram in fig. 3a in [22]). *Type 1* crystals have a mixing ratio *f* and *Type 2* crystals have a mixing ratio (1 − *f*), while the planar angle, *ϕ*, of *Type 2* crystals is a uniformly distributed random variable on the interval [0,*π*). The layer thickness values for *Cl. harengus* in [1,^22^] are assumed and are typical of the more strongly disordered animal multilayer reflectors described in §2.

We consider polarizing and polarization-insensitive reflection as two separate cases of the model. The case of a polarizing reflection occurs for a multilayer with solely *Type 1* crystals where *f* = 1. A polarizing reflection also occurs for any multilayer with isotropic crystals where *n*_o_ = *n*_e_, and we also include this isotropic limit as a reference. Models of both *Type 1* crystals and isotropic crystals correspond to a transfer matrix system with uncoupled polarization modes [47,48], and the localization length is calculated using the uncoupled transmissivity for *s*- and *p*-polarizations. The case of a polarization-insensitive reflection occurs for a mixture of *Type 1* and *Type 2* crystals and corresponds to the ‘two-crystal system’ described in [22]. The inclusion of *Type 2* crystals leads to cross reflectivity and transmissivity terms, *R_sp_*, *R_ps_*, *T_sp_*, *T_ps_*, and following the approach in [64] the localization lengths are computed using *T_s_* = *T_ss_* + *T_sp_* and *T_p_* = *T_pp_* + *T_ps_*.

### 5.2. Localization and reflectivity for a broadband polarizing animal reflector

**Figure 8.**
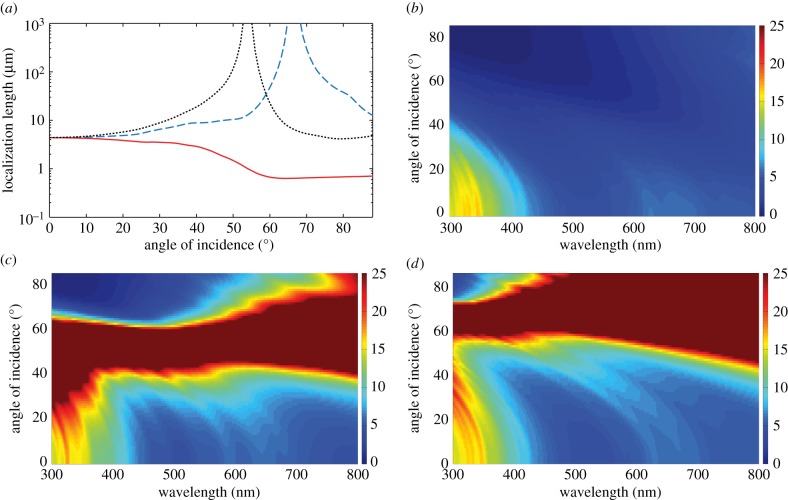
Angular and spectral properties of the localization length for a broadband polarizing animal multilayer reflector. (*a*) Angular logarithmic scale plot for *λ* = 500 nm. The red solid line is for *l_s_*(*θ*), the black dotted line is *l_p_*(*θ*) for isotropic guanine crystal layers, and the blue long dashed line is *l_p_*(*θ*) for *Type 1* guanine crystal layers. Panels (*b*)–(*d*) are two-dimensional spectral–angular plots for *l_s_*(*λ*,*θ*), *l_p_*(*λ*,*θ*) for isotropic guanine crystal layers, and *l_p_*(*λ*,*θ*) for *Type 1* guanine crystal layers, respectively. The colour-map scale for the localization length in (*b*)–(*d*), is defined on the interval [0, 25] µm, where dark red is 25 µm and dark blue is 0. All plots use the layer thickness values for *Clupea harengus* in table 1 with crystal mixing ratio *f* = 1. The simulations were averaged over 500 stack configurations.

Figure 8*a* shows the angular dependence of *l_s_* and *l_p_* at *λ* = 500 nm for multilayers with isotropic crystals and *Type 1* crystals (*f* = 1) plotted on a logarithmic scale. *l_s_* is identical in both cases and has a maximum at normal incidence, where it is approximately 4.5 µm. *l_p_* has strong angular dependence and diverges at the interfacial Brewster angles of each structure, which for the multilayer with isotropic crystals is given by Orfanidis [59]5.1
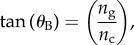
and for the multilayer with *Type 1* crystals is given by5.2
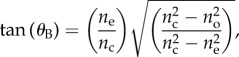
which corresponds to 54° and 67°, respectively. The divergence of the localization length for *p*-polarized light is referred to as either the ‘stochastic Brewster’ effect [60] or the ‘Brewster anomaly’ effect as it represents vectorial ‘anomalous’ behaviour from the general result that all scalar waves are localized in one dimension [49]. At these angles of incidence, *p*-polarized light is perfectly transmitted through the multilayer structure and the multiple scattering and interference required for localization to occur is inhibited [49].

Figure 8*b–d* shows corresponding two-dimensional (two-dimensional) spectral–angular colour-maps for *l_s_*(*λ*,*θ*), *l_p_*(*λ*,*θ*) for isotropic crystal layers and *l_p_*(*λ*,*θ*) for *Type 1* crystals (*f* = 1), respectively. In each plot, the colour-map scale for the localization lengths are on the interval [0,25] µm where dark red is 25 µm and dark blue is 0. Owing to the divergence in *l_p_*, the solid dark red areas in figure 8*c,d* represent values of *l_p_*(*λ*,*θ*) that are considerably greater than 25 µm. However, as the vast majority of animal reflectors in §2 are thinner than 25 µm, this length represents a suitable truncation for an animal reflector to be able to effectively localize light. Figure 8*b–d* demonstrates that for both polarizations the angular dependence of localization length at *λ* = 500 nm in figure 8*a* is typical of that which is observed across the animal-visible spectrum. However, the angular dependence of localization length is not entirely wavelength-independent, owing to the thickness disorder being weaker than the mathematically ideal strong disorder limit discussed in §4.

**Figure 9.**
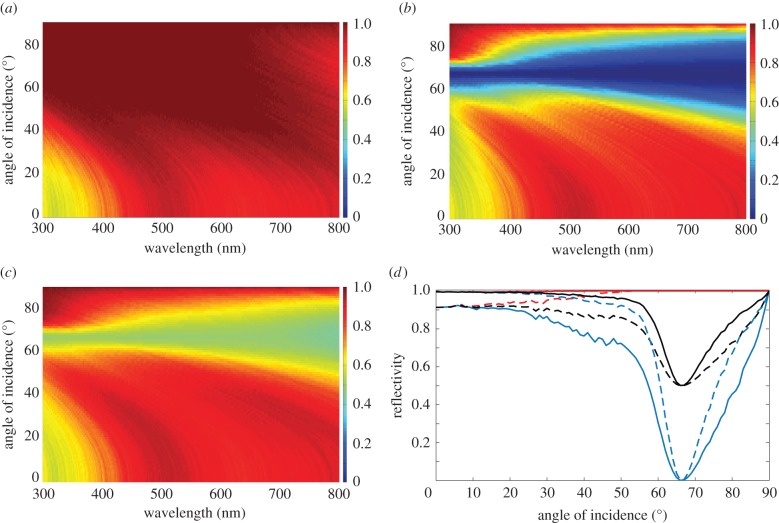
Angular and spectral properties of the reflectivity for a broadband polarizing animal multilayer reflector. (*a*)–(*c*) Spectral–angular plots for *R_s_*(*λ*,*θ*), *R_p_*(*λ*,*θ*), *R*_mean_(*λ*,*θ*) = 1*/*2(*R_s_* + *R_p_*) for *N* = 40 crystal layers and the same parameter set as figure 8. (*d*) Corresponding angular reflectivity curves for *R_s_*(*θ*) (red long dashed curve *N* = 40, red solid curve *N* = 100), *R_p_*(*θ*) (blue long dashed curve *N* = 40, blue solid curve *N* = 100), *R*_mean_(*θ*) (black long dashed curve *N* = 40, black solid curve *N* = 100) at *λ* = 500 nm. The colour-map scale for the reflectivity in (*a*)–(*c*) is defined on the interval [0, 1], where dark red is 1 dark blue is 0. The simulations were averaged over 500 stack configurations.

Two-dimensional spectral–angular reflectivity plots for *R_s_*(*λ*,*θ*) and *R_p_*(*λ*,*θ*) for *Type 1* crystals (*f* = 1) are shown in figure 9*a,b* and correspond to the localization lengths in figure 8*b*,*d*. The reflectivity plots assume *N* = 40 crystal layers, which corresponds to a total reflector length, *L*, of approximately 9.9 µm. It is clear that, for a reflector of this length, values of *l_s_* that are typically approximately 4 µm or less, are associated with *R_s_* ∼ 0.8 or greater across the majority of the animal-visible spectrum and angles of incidence. The correspondence between the divergence in *l_p_* in figure 8*d* and the drop in reflectivity for *R_p_* in the angular region of the Brewster angle, 67°, is also evident. A plot for *R_p_*(*λ*,*θ*) for isotropic crystals is not included. This produces physically analogous behaviour to *R_p_* in the birefringent model but with the *p*-polarization reflection minimum at the isotropic Brewster angle of 54°. A further feature worth noting here is that, in an analogous fashion to periodic quarter-wave reflectors [4,32], the reflectance maximum shifts towards shorter wavelengths with increasing angles of incidence.

Figure 9*c* shows the mean reflectivity, *R*_mean_ = (1*/*2)(*R_s_* + *R_p_*), calculated by averaging plots figure 9*a*,*b*. This is the reflectivity that is observed for incident unpolarized light. An important consequence of the stochastic Brewster effect is that it places a bound upon the mean reflectivity of 0.5 at Brewster's angle. This bound is clearly illustrated in figure 9*d* which shows *R_s_*(*θ*), *R_p_*(*θ*) and *R*_mean_(*θ*) at *λ* = 500 nm for *N* = 40 and 100 (the later corresponding to a reflector length approx. 24.8 µm and the number of crystals reported for *L. caudatus* in [21] and table 1).

### 5.3. Localization and reflectivity for a broadband polarization-insensitive animal multilayer reflector

**Figure 10.**
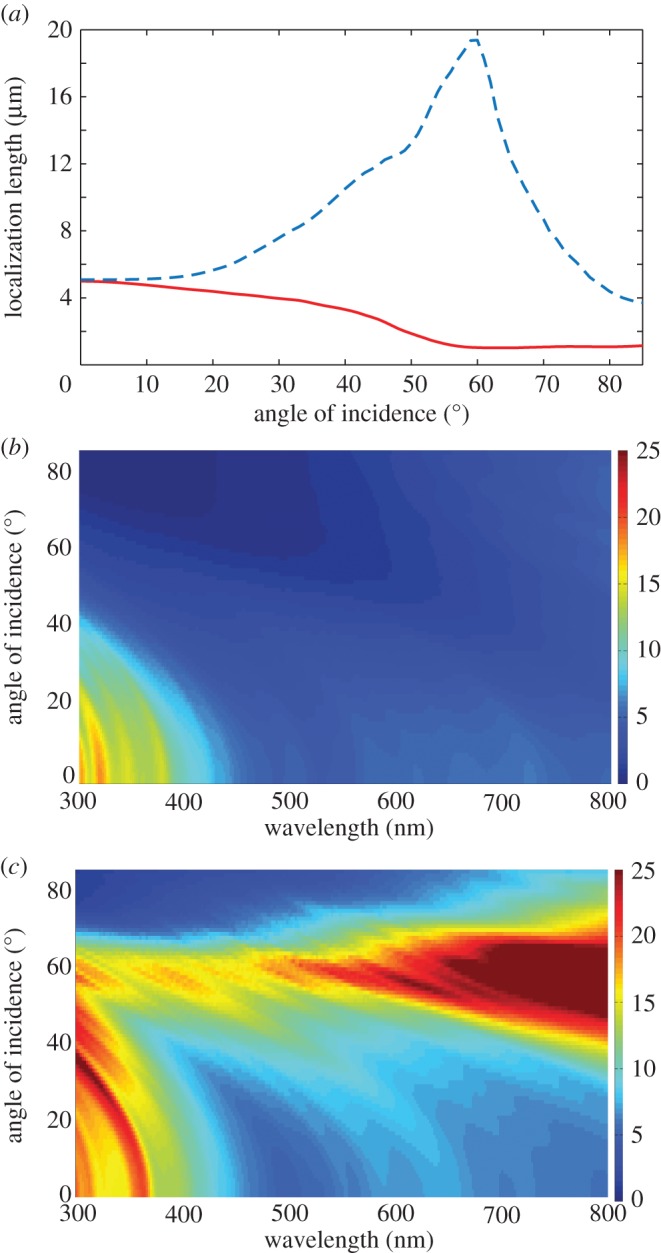
Angular and spectral properties of the localization length for a broadband polarization-insensitive animal multilayer reflector. (*a*) Angular plot for *λ* = 500 nm. The red solid line is for *l_s_*(*θ*) and the blue long dashed line is for *l_p_*(*θ*). Note that the localization lengths are an order of magnitude less than those report in figure 8*a*. (*b*,*c*) Two-dimensional spectral–angular plots for *l_s_*(*λ*,*θ*) and *l_p_*(*λ*,*θ*). The colour-map scale for the localization length in (*b*,*c*) is defined on the interval [0, 25] μm, where dark red is 25 μm and dark blue is 0. All plots use the layer thickness values for *Cl. harengus* in table 1 with crystal mixing ratio *f* = 0.75. The simulations were averaged over 500 stack configurations.

Figure 10*a* shows *l_s_*(*θ*) and *l_p_*(*θ*) at *λ* = 500 nm for a multilayer with a mixture of *Type 1* and *Type 2* crystals with a mixing ratio *f* = 0.75 (as modelled for *Cl. harengus* in [22]). The angular dependance of *l_s_*(*θ*) in figure 10*a* is similar to the equivalent plot for *Type 1* crystals (figure 8*a*). However, the behaviour for *l_p_*(*θ*) is very different for the two-crystal system, with *l_p_* less than 20 µm over all angles of incidence. (Note: figure 10*a* is on a linear scale and figure 8*a* is on a logarithmic scale.) Figure 10*b,c* shows corresponding two-dimensional spectral–angular colour-maps for *l_s_*(*λ*,*θ*) and *l_p_*(*λ*,*θ*), respectively. Values of *l_p_* less than 25 µm over all angles of incidence are observed over the spectral region approximately 350–600 nm in figure 10*c*, demonstrating a broadband ‘suppression’ of the stochastic Brewster effect that is very different from that observed in isotropic crystal stacks (figures 8 and 9).

The polarization-insensitive localization of light that occurs for the isotropic (cytoplasm)–birefringent (guanine) multilayer in figure 10 does not occur in isotropic random stack structures [49,56,60] and represents an optically novel behaviour that had not previously been described in the physics literature [20]. The physical origin is primarily due to the interfacial Brewster angles (i.e. the interfacial *p*-polarization reflection minima) of *Type 1* and *Type 2* crystals having a much wider angular separation than is possible in isotropic random stack systems (the Brewster angles of *Type 2* crystals occur at 33° and 54°) [22]. This interpretation is supported by mathematical analysis in [20], which obtains an analytical expression for *l_p_* for a closely analogous isotropic–birefringent stack system in terms of isotropic–birefringent Fresnel reflection amplitudes [48,65].

**Figure 11.**
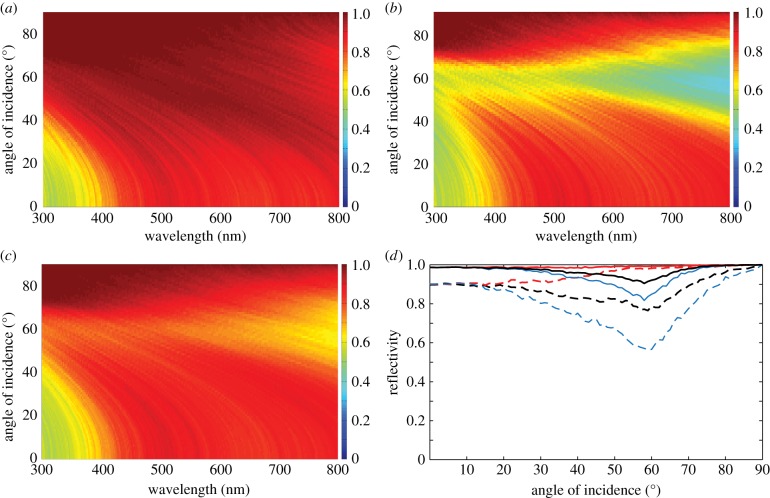
Angular and spectral properties of the reflectivity for a broadband polarization-insensitive animal multilayer reflector. (*a*)–(*c*) Spectral–angular plots for *R_s_*(*λ*,*θ*), *R_p_*(*λ*,*θ*), *R*_mean_(*λ*,*θ*) = (1/2)(*R_s_* + *R_p_*) for the thickness parameters for *Cl. harengus* in table 1 with crystal mixing ratio *f* = 0.75 and *N* = 40 crystal layers. The colour-map scale for the reflectivity is defined on the interval [0, 1], where dark red is 1 and dark blue is 0. (*d*) Angular reflectivity curves at *λ* = 500 nm for *R_s_*(*θ*) (red long dashed curve *N* = 40, red solid curve *N* = 100), *R_p_*(*θ*) (blue long dashed curve *N* = 40, blue solid curve *N* = 100), *R*_mean_(*θ*) (black long dashed curve *N* = 40, black solid curve *N* = 100). The simulations were averaged over 500 stack configurations.

Reflectivity for the two-crystal system is calculated using *R_s_* = *R_ss_* + *R_sp_* and *R_p_* = *R_pp_* + *R_ps_*, with the mean reflectivity *R*_mean_ = 1*/*2(*R_s_* + *R_p_*) as before. Two-dimensional spectral–angular reflectivity plots for *R_s_*(*λ*,*θ*) and *R_p_*(*λ*,*θ*), and *R*_mean_(*λ*,*θ*) for *N* = 40 crystal layers are shown in figure 11*a–c*, respectively, with figure 11*d* being an angular plot at *λ* = 500 nm for *N* = 40 and 100 crystal layers, respectively. These plots directly follow the previous presentation of the reflectivity for the equivalent broadband polarizing reflector (figure 9). The non-divergent localization length for *p*-polarized light in figure 10*a*,*c*, means that the mean reflectivity now has a theoretical upper bound of 1. Figure 11*d*, illustrates that for *λ* = 500 nm, the reflector with two types of crystal has *R_p_* greater than 0.5 and 0.8 over all angles of incidence for *N* = 40 and 100 crystal layers, respectively. Correspondingly, *R*_mean_ is high, and approximately ‘flat’, over all angles of incidence. The two-dimensional spectral–angular reflectivity plots (figure 11*a,c*) demonstrate that the high reflection region for both polarizations is correlated with the regions of lower localization length in figure 10*b*,*c*. Increasing the number of crystal layers to *N* = 100 increases the mean reflectivity to being approximately 0.9 or greater over all angles of incidence.

## 6. Summary

The theory of Anderson localization in one dimension applies to models of disordered animal multilayer reflectors and gives a general framework of how the spectral and polarization properties of reflectivity are controlled by thickness disorder and birefringence, respectively. Of particular importance is the general exponential decay in transmissivity, which arises purely from a coherent scattering process. This enables the localization length to be calculated for these systems.

Over the past 40 years, the ‘quarter-wave stack’ model of animal reflectors has proved to be a valuable tool for vision ecologists in establishing a link between the reflectivity properties of periodic animal multilayer reflectors and visually guided animal behaviour [4,14,29,66]. The localization framework presented in this paper provides an extension to this method as a way of understanding the optical characteristics of animal multilayer reflectors with varying degrees of disorder. Localization is itself the physical foundation for the optics of these structures and the localization length could be described as a universal property under selection in relation to biological function. Furthermore, the calculation of the localization length for disordered animal multilayer reflectors could potentially play a similar role to the calculation of the band-gap for periodic animal reflectors and higher dimensional animal photonic crystals and in both cases this additional ‘propagation information’ enables regions of high reflectivity to be predicted and explained in terms of the propagation of light.

Control over the polarization properties of reflection has been a topic of recent debate in ecology of vision circles, particularly in the context of camouflage in silvery marine fish [12,22,67]. For the ideal case of an axially symmetric underwater light field, non-polarizing reflectivity over all angles of incidence provides maximum reflectivity and therefore optimal concealment [22]. The polarization-insensitive localization of light for the model of *Cl. harengus* and *Sa. pilchardus* in figure 10 provides an explanation for how these fish produce polarization-insensitive reflectively over a broad wavelength regime. The two-crystal mechanism (which can, in principle, produce 100% mean reflectivity over all angles of incidence if spatial averaging of the reflectivity is assumed [22]) is most likely to enable the reflections from the fish better match both the background intensity and polarization of the underwater light field than a polarizing reflector (which can only produce 50% mean reflectivity at Brewster's angle). The cost of such highly reflective, non-polarizing broadband multilayer stacks, includes the need for the control of the layer thickness disorder and a defined packing fraction of the two types of guanine crystal. Biologically, such costs are difficult to quantify without a detailed knowledge of the metabolic processes underlying the stack assembly and maintenance. They are, however, likely to be small compared with the costs of failed concealment.

While the numerical calculations in this paper used guanine–cytoplasm reflectors as a model system, the localization perspective also applies to random stack models of protein–cytoplasm reflectors in cephalopods [26,27,30]. The coloured protein–cytoplasm reflectors in squid iridophores (*Loligo pealeii* [26] and *Doryteuthis opalescens* [27]) have *σ*_R_ ∼ 0.1–0.2 for both layer types and are therefore similar to the more weakly disordered guanine–cytoplasm systems. However, silvery ‘spindle’ protein structures around the eye of the squid *Loligo forbesi* (which behave analogously to a one-dimensional multilayer system) are highly disordered and have *σ*_R_ ∼ 0.6 for both layer types [30]. Transfer matrix models of protein–cytoplasm reflectors assume that the refractive index of the protein platelets is 1.56 [3,4,26,30]. Relative to the higher refractive index guanine–cytoplasm reflectors, this would result in the localization length being longer for a given level of thickness disorder and the percentage reflectivity for a given number of stack layers being lower. Random stack models of dielectrically isotropic protein–cytoplasm reflectors cannot control the polarization properties of reflected light and are always predicted to fully polarize at Brewster's angle.

The localization perspective provided in this paper could also be used as starting point to revisit theoretical models of some past studies of animal multilayer reflectors. One potential area to examine would be the effect of correlated layer thickness disorder upon the reflectivity. Correlated layer thickness is reported in the study by Levy-Lior *et al.* [24] for guanine–cytoplasm reflectors in the iridophores of *Cy. carpio* where it is estimated that there is a nearest neighbour correlation of approximately 60% in the spacing of the cytoplasm gaps between crystals within one iridophore cell. It is known in the physics literature that correlated thickness disorder has the effect of diminishing the underlying band-gap structure more effectively than equivalent uncorrelated thickness disorder [68]. Consequently, the presence of correlated thickness disorder would broaden the spectral reflection bandwidth more effectively for a given reflector length and may therefore potentially be an adaptation for animal multilayer reflectors that require a broadband reflection with a minimal number of layers and minimum energetic cost.
